# On certain new refinements of Finsler-Hadwiger inequalities

**DOI:** 10.1186/s13660-017-1356-5

**Published:** 2017-04-18

**Authors:** Omran Kouba

**Affiliations:** Department of Mathematics, Higher Institute for Applied Sciences and Technology, P.O. Box 31983, Damascus, Syria

**Keywords:** 11B68, triangle inequalities, Finsler-Hadwiger inequality

## Abstract

Several refinements of the Finsler-Hadwiger inequality and its reverse in the triangle are discussed. A new one parameter family of Finsler-Hadwiger inequalities and their reverses are proved. This allows us to obtain new bounds for the sum of the squares of the side lengths of a triangle in terms of other elements in the triangle. Finally, these new bounds are compared to known ones.

## Introduction

In a triangle $\triangle ABC$ the angle measures are referred to as *A*, *B* and *C* and the lengths of the sides *BC*, *CA* and *AB* are denoted by *a*, *b* and *c*, respectively. As is customary, the circumradius, the inradius, the semiperimeter and the area of the triangle are denoted by *R*, *r*, *s* and *F*, respectively. The deviation of the triangle from being equilateral is measured by the quantity

$$Q=(a-b)^{2}+(b-c)^{2}+(c-a)^{2}. $$

With the above notation, the celebrated Weitzenböck inequality [1] states that

1.1 $$ a^{2}+b^{2}+c^{2}\ge4\sqrt{3}F. $$

This inequality was published in 1919, but the authors in [2] traced it back to 1897 where it was proposed as Problem 273 in the Romanian magazine Gazeta Matematică (III(2), p.52) by Ionescu. So they proposed to call it the ‘Ionescu-Weitzenböck inequality’. At least five distinct proofs of (1.1) could be found in [3] and [4].

The Ionescu-Weitzenbök inequality was later refined by Finsler and Hadwiger (see [5, 6]), to give birth to the Hadwiger-Finsler inequality,

1.2 $$ a^{2}+b^{2}+c^{2}\ge Q+ 4\sqrt{3}F, $$

and its reverse,

1.3 $$ 3 Q+ 4\sqrt{3}F\ge a^{2}+b^{2}+c^{2}, $$

with equality in each of these inequalities occurring if and only if the triangle is equilateral. In [7], pp.170-174, 11 proofs of (1.1) and several proofs of (1.2) are presented. In [8] we find the following equivalent form of (1.2):

1.4 $$ 2(ab+bc+ac)-\bigl(a^{2}+b^{2}+c^{2} \bigr)\ge 4\sqrt{3} F, $$

and another chain of refinements of (1.1), namely

1.5 $$ a^{2}+b^{2}+c^{2}\ge ab+bc+ca \ge3(abc)^{2/3}\ge4\sqrt{3}F. $$

Note that the rightmost inequality in this chain is called ‘the Polya-Szegö inequality’ (see [9], p.161, Problem 17.1 and [10], p.116).

It is proved in [11], Theorem 3.1, that inequalities (1.2) and (1.3) are optimal in the sense that if for some real numbers *λ* and *μ*, we have

1.6 $$ \lambda Q+ 4\sqrt{3}F\ge a^{2}+b^{2}+c^{2}\ge\mu Q+ 4\sqrt{3}F $$

in any triangle $\triangle ABC$, then $\lambda\ge3$ and $\mu\le1$. However, it is proved in [12, 13] that the least *λ* for which the upper inequality holds in any *non-obtuse* triangle is $(2-\sqrt{3})(3+2\sqrt{2})\approx1.5617$.

Surprisingly, it was noted in [14] that the Ionescu-Weitzenböck inequality (1.1) is equivalent to Finsler-Hadwiger inequality (1.2) by showing that the second follows from the first by applying the first to another triangle.

## Results and discussion

In this paper we consider refinements of (1.2) and (1.3) in the following form:

2.1 $$ 3Q+4\sqrt{3} F\psi^{+} \biggl(\frac{2r}{R} \biggr) \ge a^{2}+b^{2}+c^{2} \ge Q+4\sqrt{3}F \psi^{-} \biggl(\frac{2r}{R} \biggr), $$

where $\psi^{-}$ and $\psi^{+}$ are functions defined on $[0,1]$, such that $\psi^{-}(1)=\psi^{+}(1)=1$ and $\psi^{+}(t)<1<\psi^{-}(t)$ for $0\le t<1$. Recall that by Euler’s inequality we have $2r\le R$ with equality if and only if $\triangle ABC$ is equilateral. We seek non-trivial $\psi^{-}$ and $\psi^{+}$ but as beautiful and simple as possible, clearly this requirement is not too mathematical, but I think no one would recall an ugly complicated inequality even if it is very sharp. I will let the reader be the judge on this statement.

In [15] the authors show that one can take $\psi^{-} =\psi_{0}^{-} $ where

2.2 $$ \psi_{0}^{-}(t)=\sqrt{\frac{32-5t}{24+3t}} =\sqrt{\frac{4-t}{3}- \frac{t(1-t)}{24+3t}}. $$

This lower bound was later improved in [16] where the authors proved using the Garfunkel-Bankoff inequality [17, 18] that one can take $\psi^{-} =\psi_{1}^{-}$ where

2.3 $$ \psi_{1}^{-}(t)=\sqrt{\frac{4-t}{3}}, $$

which is stronger than $\psi_{0}^{-}$ and more beautiful! In fact, we will see that this strengthened inequality is equivalent to Kooi’s inequality [19] (see also [20], Inequality 5.7).

But what about the upper bound $\psi^{+}$? To the best of the knowledge of the author, it seems that no refinement better than the constant $\psi^{+}_{0}\equiv1$ is known or has been published. In this note we will prove that $\psi^{+}=\psi_{1}^{+}$ with $\psi_{1}^{+}(t)=\sqrt{t}$ is a refinement of the reverse Finsler-Hadwiger inequality, and we will also provide an alternative proof (different from the one given in [16]) of the lower bound with $\psi_{1}^{-}$.

Therefore, our first result concerning the refinement of Finsler-Hadwiger inequality and its reverse (Theorem 3.6) will be the following:

2.4 $$ 3Q+4F \sqrt{\frac{6r}{R}} \ge a^{2}+b^{2}+c^{2} \ge Q+4 F \sqrt{4-\frac{2r}{R}}. $$

Further, in Theorem 3.7, the next alternative ‘reverse Finsler-Hadwiger inequality’ is proposed,

2.5 $$ Q+4F \sqrt{1+\frac{R}{r}} \ge a^{2}+b^{2}+c^{2}. $$

These two reverses are then compared and it is proved in Corollary 3.9 that we have the following chain of inequalities:

2.6 $$ 3Q+4F \sqrt{\frac{6r}{R}} \ge Q+4F \sqrt{1+\frac{R}{r}} \ge a^{2}+b^{2}+c^{2}. $$

In the final step of this investigation we consider a one parameter family of inequalities, namely

2.7 $$ (1+2\lambda)Q+4F \biggl((1-\lambda)\sqrt{1+\frac{R}{r}}+\lambda \sqrt{ \frac{6r}{R}} \biggr)\ge a^{2}+b^{2}+c^{2} $$

and we prove in Corollary 3.10 that it holds for $\lambda\ge0$, its reverse holds for $\lambda\le-\frac{17+8\sqrt{6}}{95}$ and that the constants 0 and $-\frac{17+8\sqrt{6}}{95}$ are the best possible.

## Theorems and proofs

The main tool in our proofs is the fundamental inequality in the triangle. This inequality has a long history, the reader may consult [21] or [22], Chapter 1, for more information.

### Theorem 3.1

The fundamental inequality

*Consider three positive numbers* *s*, *r* *and* *R* *with* $2r\le R$. *Then* *s*, *r* *and* *R* *are*, *respectively*, *the semiperimeter*, *the inradius and the circumradius of a triangle if and only if*

3.1 $$ \phi \biggl(\frac{2r}{R} \biggr)\le\frac{s^{2}}{R^{2}} \le\Phi \biggl( \frac{2r}{R} \biggr), $$

*where*

3.2 $$\begin{aligned}& \phi(t) = 2+5t-\frac{t^{2}}{4}-2(1-t)^{3/2}, \end{aligned}$$

3.3 $$\begin{aligned}& \Phi(t) = 2+5t-\frac{t^{2}}{4}+2(1-t)^{3/2}. \end{aligned}$$

*Moreover*, *the upper equality holds in* (3.1) *if and only if the triangle is isosceles and the radian measure of its apex is larger or equal to* $\pi/3$. *Similarly*, *the lower equality holds in* (3.1) *if and only if the triangle is isosceles and the radian measure of its apex is smaller or equal to* $\pi/3$.

We will use also several algebraic inequalities that are gathered in the next two lemmas.

### Lemma 3.2

*The following inequalities hold*.

(i) *For* $t\in[0,1]$ *we have*
  3.4 $$ 2+5t-\frac{t^{2}}{4}+2(1-t)^{3/2}\le\frac{(8+t)^{2}}{4(4-t)}. $$
(ii) *For* $t\in[0,1]$ *we have*
  3.5 $$ 2+5t-\frac{t^{2}}{4}-2(1-t)^{3/2}\ge\frac{t ( \sqrt{128+16 t+3t^{2}}-\sqrt{3} t )^{2}}{16}. $$
(iii) *For* $t\in[0,1]$ *we have*
  3.6 $$ 2+5t-\frac{t^{2}}{4}-2(1-t)^{3/2} \ge\frac{ t(8+t)^{2}}{4(2+t)}. $$

*Moreover*, *equality holds in any of the above inequalities if and only if* $t\in\{0,1\}$.

### Proof

(i) Let

$$F(t)= \frac{(8+t)^{2}}{4(4-t)}- \biggl(2+5t-\frac{t^{2}}{4}+2(1-t)\sqrt {1-t} \biggr) $$

then it is readily seen that

$$\begin{aligned} F(t) &=\frac{1-t}{4(4-t)} \bigl(32-24t+t^{2}-8(4-t)\sqrt{1-t} \bigr) \\ &=\frac{1-t}{4(4-t)} (4-4\sqrt{1-t}-t )^{2} \\ &=\frac{(1-t)t^{2}}{4(4-t)} \biggl(\frac{4}{1+\sqrt{1-t}}-1 \biggr)^{2}\ge0, \end{aligned}$$

and (3.4) follows.

To prove (ii) we note that, after expanding the square, the proposed inequality is equivalent to $G(t)\ge0$ for $t\in[0,1]$ where

$$G(t)=2-2\sqrt{(1-t)^{3}}-3t-\frac{5}{4}t^{2}- \frac{3}{8}t^{3}+\frac {t^{2}}{8}\sqrt{384+48t+9t^{2}}. $$

Now, for $t\in[0,1]$ we have

$$ (1-\sqrt{1-t})^{2}(1-t)=2-2(1-t)\sqrt{1-t}-3t+t^{2}, $$

hence

$$\begin{aligned} G(t)-(1-\sqrt{1-t})^{2}(1-t) &=\frac{t^{2}}{8} \bigl( \sqrt{384+48t+9t^{2}}-3t-18 \bigr) \\ &=\frac{15t^{2}(1-t)}{2\sqrt{384+48t+9t^{2}}+6(t+6)}. \end{aligned}$$

Finally

$$ G(t)=t^{2}(1-t) \biggl(\frac{1}{(1+\sqrt{1-t})^{2}}+ \frac{15}{2\sqrt{384+48t+9t^{2}}+6(t+6)} \biggr) \ge0. $$

This concludes the proof of (3.5).

(iii) According to (ii) we only have to prove that

3.7 $$ \bigl(\sqrt{128+16 t+3t^{2}}-\sqrt{3} t \bigr)^{2} \ge\frac{ 4(8+t)^{2}}{2+t}. $$

Again, expanding the square we see that the proposed inequality is equivalent to $H(t)\ge0$ for $t\in[0,1]$ where

$$H(t)=128+16t+6t^{2}-\frac{4(8+t)^{2}}{2+t}-2t\sqrt{384+48t+9t^{2}}. $$

But

$$\begin{aligned} H(t)&=\frac{ 96 t+24t^{2}+6t^{3}}{2+t}-2t\sqrt{384+48t+9t^{2}} \\ &=2t \biggl(\frac{ 48 +12t+3t^{2}}{2+t}-\sqrt{384+48t+9t^{2}} \biggr) \\ &=2t \biggl(\frac{ 6-9t+3t^{2}}{2+t}+21-\sqrt{384+48t+9t^{2}} \biggr) \\ &=2t \biggl(\frac{ 3(1-t)(2-t)}{2+t}+\frac{57-48t-9t^{2}}{21+\sqrt {384+48t+9t^{2}}} \biggr) \\ &=6t(1-t) \biggl(\frac{ 2-t}{2+t}+\frac{19+3t}{21+\sqrt {384+48t+9t^{2}}} \biggr)\ge0, \end{aligned}$$

and (3.6) follows. □

### Remark 3.3

The proof shows that the ‘ugly’ inequality (3.5) is stronger than (3.6).

### Proposition 3.4

*Let the semiperimeter*, *the inradius and the circumradius of a triangle be denoted by* *s*, *r* *and* *R*, *respectively*. *Then*

3.8 $$\begin{aligned}& s\sqrt{4 -\frac{2r}{R}} \le4R+r, \end{aligned}$$

3.9 $$\begin{aligned}& s\sqrt{1+\frac{R}{r}} \ge4R+r, \end{aligned}$$

3.10 $$\begin{aligned}& 4r(4R+r) \le sr\sqrt{\frac{6r}{R}}+s^{2}. \end{aligned}$$

*Moreover*, *equality holds in any of the above inequalities if and only if the triangle is equilateral*.

### Proof

The well-known Euler inequality states that $2r\le R$ (with equality if and only if the triangle is equilateral) so we may apply (3.4) from Lemma 3.2 with $t=2r/R$ and then multiply both sides of the resulting inequality by $R^{2}$ to obtain

3.11 $$ 2R^{2}+10rR-r^{2}+2\sqrt{R(R-2r)^{3}}\le \frac{(r+4R)^{2}R}{4R-2r}. $$

Combining this with Theorem 3.1 we get

$$ s^{2}\le\frac{(r+4R)^{2}R}{4R-2r}, $$

or equivalently

3.12 $$ s\sqrt{4-\frac{2r}{R}}\le r+4R. $$

Similarly, applying (3.6) from Lemma 3.2 with $t=2r/R$, multiplying both sides of the resulting inequality by $R^{2}$, and finally making use of Theorem 3.1 we obtain

$$s^{2}\ge\frac{r(4R+r)^{2}}{R+r}, $$

which is equivalent to (3.9).

Doing the same manipulation with (3.5) we get

3.13 $$ 2R^{2}+10rR-r^{2}-2\sqrt{R(R-2r)^{3}}\ge \frac{r ( \sqrt{32R^{2}+8 rR+3r^{2}}-\sqrt{3} r )^{2}}{2R}, $$

and according to Theorem 3.1 we obtain

$$ s^{2}\ge \frac{r ( \sqrt{32R^{2}+8 rR+3r^{2}}-\sqrt{3} r )^{2}}{2R}, $$

or equivalently

$$ s\sqrt{\frac{2R}{r}}+\sqrt{3}r\ge \sqrt{32R^{2}+8 rR+3r^{2}}. $$

Finally, squaring and rearranging we get (3.10). □

### Remark 3.5

Inequalities (3.8) and (3.9) are not new, see [23], Inequality 2.15, and particularly, (3.8) is Kooi’s inequality [19].

Now we are ready to give an alternative proof of the refinement of Finsler-Hadwiger inequality given in [16] and to present our refinement of its reverse, as announced in the Introduction.

### Theorem 3.6

*In a triangle* $\triangle ABC$ *let the inradius*, *the circumradius*, *the area and the lengths of sides opposite to angles* *A*, *B* *and* *C* *be denoted by* *r*, *R*, *F*, *a*, *b* *and* *c*, *respectively*. *If* $Q=(a-b)^{2}+(b-c)^{2}+(c-a)^{2}$ *then the following two inequalities hold*:

3.14 $$\begin{aligned}& a^{2}+b^{2}+c^{2} \ge Q+4F\sqrt{4- \frac{2r}{R}} , \end{aligned}$$

3.15 $$\begin{aligned}& a^{2}+b^{2}+c^{2} \le3Q+4F\sqrt{ \frac{6r}{R}}. \end{aligned}$$

*Moreover*, *equality holds in any of the above inequalities if and only if the triangle is equilateral*.

### Proof

Using Heron’s formula and the facts that $F=rs$ and $abc=4RF$ we obtain

$$ r^{2}s=\frac{F^{2}}{s}=(s-a) (s-b) (s-c)=-s^{3}+s(ab+bc+ca)-4Rrs. $$

Hence

3.16 $$ ab+bc+ca=s^{2}+r^{2}+4Rr. $$

Moreover, because $4s^{2}=a^{2}+b^{2}+c^{2}+2(ab+bc+ca)$ we also conclude that

3.17 $$ a^{2}+b^{2}+c^{2}=2 \bigl(s^{2}-r^{2}-4Rr\bigr). $$

It follows that

3.18 $$\begin{aligned} a^{2}+b^{2}+c^{2}-Q&=2(ab+bc+ca)-a^{2}-b^{2}-c^{2} \\ &=4r^{2}+16Rr=4r(r+4R). \end{aligned}$$

So (3.14) follows from (3.8) by multiplying both sides by 4*r* and using (3.18).

Similarly, from (3.16) and (3.17) we conclude that

3.19 $$\begin{aligned} a^{2}+b^{2}+c^{2}-3Q&= 6(ab+bc+ca)-5 \bigl(a^{2}+b^{2}+c^{2}\bigr) \\ &=64Rr+16r^{2}-4s^{2}. \end{aligned}$$

So (3.15) follows from (3.10). □

In the next result we provide an alternative ‘reverse Finsler-Hadwiger inequality’.

### Theorem 3.7

*Let* $\triangle ABC$ *be a triangle*, *and let the lengths of sides opposite to angles* *A*, *B* *and* *C* *be denoted by* *a*, *b* *and* *c*, *respectively*. *If* *F* *represents the area of* $\triangle ABC$, *and if* $Q=(a-b)^{2}+(b-c)^{2}+(c-a)^{2}$ *then the following inequality holds*:

$$ a^{2}+b^{2}+c^{2}\le Q+4F\sqrt{1+ \frac{R}{r}}, $$

*with equality if and only if the triangle is equilateral*.

### Proof

Indeed, this follows from

$$a^{2}+b^{2}+c^{2}-Q=4r(r+4R)\le4rs\sqrt{1+ \frac{R}{r}} =4F\sqrt{1+\frac{R}{r}}, $$

where we used (3.9) from Proposition 3.4 and (3.17). □

In fact Theorems 3.6 and 3.7 yield two different upper bounds for the sum of squares of the side lengths of a triangle in terms of its area, and they are both candidates to be called the ‘reverse Finsler-Hadwiger inequality’. A legitimate question is the following: Are these two bounds comparable? Some numerical experimentation shows that the upper bound given in Theorem 3.7 is better than the corresponding upper bound given in Theorem 3.6. This suggests that the inequality

$$ 2F\sqrt{1+\frac{R}{r}}\le Q+2F\sqrt{\frac{6r}{R}} $$

is valid in any triangle. Rearranging this inequality we see that it is equivalent to

$$ rs\sqrt{1+\frac{R}{r}}\le a^{2}+b^{2}+c^{2}-ab-bc-ca+rs \sqrt{\frac{6r}{R}} $$

or, by (3.16) and (3.17), to

$$ 3r(r+4R)+rs\sqrt{1+\frac{R}{r}}\le s^{2}+rs\sqrt{ \frac{6r}{R}}. $$

Writing this as

$$4rs\sqrt{1+\frac{R}{r}}-rs\sqrt{\frac{6r}{R}}-s^{2}\le3r \biggl(s\sqrt{1+\frac{R}{r}}-4R-r \biggr) $$

leads to the following question what the least constant *μ* is such that the following inequality holds:

$$ 4rs\sqrt{1+\frac{R}{r}}-rs\sqrt{\frac{6r}{R}}-s^{2}\le4\mu r \biggl(s\sqrt{1+\frac{R}{r}}-4R-r \biggr) . $$

Indeed, this inequality holds clearly for $\mu\ge1$ because we have $s\sqrt{1+\frac{R}{r}}\ge4R+r$, and it does hold for $\mu=1$ according to Proposition 3.4. Testing it with a triangle $\triangle ABC$ having side lengths $a=1$, $b=c=t$ with $t>1/2$ but near $1/2$ shows that a necessary condition for such an inequality to hold is $\mu\ge\lambda=5-2\sqrt{6}=\frac{1}{5+2\sqrt{6}}\approx0.10102$. This observation is confirmed in the next theorem.

### Theorem 3.8

*Let* $\triangle ABC$ *be a triangle*, *and let* *r*, *R* *and* *s* *represent the inradius*, *the circumradius and the semiperimeter of* $\triangle ABC$, *respectively*. *Then the following inequality holds*:

$$ 4rs\sqrt{1+\frac{R}{r}}-rs\sqrt{\frac{6r}{R}}-s^{2}\le4\mu r \biggl(s\sqrt{1+\frac{R}{r}}-4R-r \biggr) $$

*for all* $\mu\ge\lambda=5-2\sqrt{6}=\frac{1}{5+2\sqrt{6}}$, *with equality if and only if the triangle is equilateral*.

### Proof

The previous discussion shows that it is enough to prove the inequality for $\mu=\lambda$. Note that the proposed inequality is equivalent to the fact that *s* is larger than the positive root of a second degree polynomial, that is,

$$\frac{s}{r}\ge 2(1-\lambda)\sqrt{1+\frac{R}{r}}-\frac{1}{2} \sqrt{\frac{6r}{R}}+\sqrt { \biggl(2(1-\lambda)\sqrt{1+\frac{R}{r}}- \sqrt{\frac {3r}{2R}} \biggr)^{2}+4\lambda \biggl(1+ \frac{4R}{r} \biggr) } $$

and according to Theorem 3.1, for a given *r* and *R*, the smallest possible value of *s* is attained when $\triangle ABC$ is isosceles. Therefore, it is enough to prove the proposed inequality for isosceles triangles.

Now, consider an isosceles triangle $\triangle ABC$. Since the desired inequality is homogeneous we may suppose that the side lengths are $a=1$ and $b=c=1/(2x)$ with $0< x<1$. In this case we have

$$ s=\frac{1+x}{2x},\qquad r=\frac{1}{2}\sqrt{\frac{1-x}{1+x}},\qquad R=\frac{1}{4x\sqrt{1-x^{2}}}. $$

Let $f(x)$ be defined for $x\in(0,1)$ by

$$ f(x)=s^{2}+rs\sqrt{\frac{6r}{R}}-4(1-\lambda)rs\sqrt{1+ \frac {R}{r}}-4\lambda r(r+4R). $$

That is,

$$\begin{aligned} f(x) =&\frac{1}{4x^{2}} \bigl((x+1)^{2}+2 (1-x) x \sqrt{3x (x+1)} \\ &{}-4(\sqrt{6}-2) \sqrt{2x \bigl(1+3x-2 x^{3} \bigr)}-4(5-2\sqrt{6}) x(2-x) \bigr). \end{aligned}$$

We can arrange this as follows:

$$\begin{aligned} f(x) =&\frac{1}{4x^{2}} \bigl((21-8\sqrt{6}) (1-x)^{2}+2 (1-x) \bigl(x \sqrt{3x (x+1)}-\sqrt{6} \bigr) \\ &{}+2(\sqrt{6}-2) \bigl(5-x-2\sqrt{2x \bigl(1+3x-2 x^{3} \bigr)} \bigr) \bigr) \\ =&\frac{(1-x)^{2}}{4x^{2}} \biggl(21-8\sqrt{6}+2\frac{ x \sqrt{3x (x+1)}-\sqrt{6} }{1-x} \\ &{}+2(\sqrt{6}-2)\frac{ 5-x-2\sqrt{2x (1+3x-2 x^{3} )}}{(1-x)^{2}} \biggr) \\ =&\frac{(1-x)^{2}}{4x^{2}} \biggl(5 +8(\sqrt{6}-2)x-2 \frac{ \sqrt{6}- x \sqrt{3x (x+1)} }{1-x} \\ &{}+2(\sqrt{6}-2) \biggl(\frac{ 5-x-2\sqrt{2x (1+3x-2 x^{3} )}}{(1-x)^{2}}-4(1+x) \biggr) \biggr) \\ =&\frac{(1-x)^{2}}{4x^{2}}\bigl(g(x)+2(\sqrt{6}-2)h(x)\bigr), \end{aligned}$$

where

$$\begin{aligned} \begin{aligned} &g(x) =5 +8(\sqrt{6}-2)x-2 \frac{ \sqrt{6}- x \sqrt{3x (x+1)} }{1-x}, \\ &h(x) = \frac{ 5-x-2\sqrt{2x (1+3x-2 x^{3} )}}{(1-x)^{2}}-4(1+x). \end{aligned} \end{aligned}$$

Now, in *g* we consider $u=\sqrt{\frac{1+x}{x}}$ as a new variable so that $x=\frac{1}{u^{2}-1}$ for $u>\sqrt{2}$. We have

$$\begin{aligned} g \biggl(\frac{1}{u^{2}-1} \biggr)&= 5 +\frac{8(\sqrt{6}-2)}{u^{2}-1}-2\sqrt{3} \frac{ \sqrt{2}(u^{2}-1)^{2}- \sqrt{3}u }{(u^{2}-1)(u^{2}-2)} \\ &=\frac{P(u)}{(u^{2}-1)(u^{2}-2)} \end{aligned}$$

with

$$P(u)=(5-2\sqrt{6})u^{4}-(31-12\sqrt{6})u^{2}+2 \sqrt{3}u+42-18\sqrt{6}. $$

Noting that $P(\sqrt{2})=P(\sqrt{3})=P'(\sqrt{3})=0$ we conclude that *P* factors as follows:

$$P(u)=(5-2\sqrt{6}) (u-\sqrt{2}) (u-\sqrt{3})^{2}(u+\sqrt{2}+2 \sqrt{3}). $$

Thus

$$g \biggl(\frac{1}{u^{2}-1} \biggr)= \lambda\frac{ (u-\sqrt{3})^{2}(u+\sqrt{2}+2\sqrt{3})}{(u^{2}-1)(u+\sqrt{2})}. $$

Or

$$g(x)= \lambda\frac{ (2x-1)^{2}(\sqrt{1+x}+\sqrt{2x}+2\sqrt{3x})}{ (\sqrt{x+1}+\sqrt {2x})(\sqrt{1+x}+\sqrt{3x})^{2}}, $$

which is clearly positive for $x\in(0,1)$ and vanishes only if $x=1/2$. On the other hand

$$\begin{aligned} (1-x)^{2}h(x)&=1+3x+4x^{2}(1-x)-2\sqrt{2x \bigl(1+3x-2x^{3}\bigr)} \\ &= \frac{(1+3x+4x^{2}(1-x))^{2}-8x(1+3x-2x^{3})}{ 1+3x+4x^{2}(1-x)+2\sqrt{2x(1+3x-2x^{3})}} \\ &=\frac{(1-x-4x^{2}+4x^{3})^{2}}{ 1+3x+4x^{2}(1-x)+2\sqrt{2x(1+3x-2x^{3})}} \\ &=\frac{(1-x)^{2}(2x-1)^{2}(2x+1)^{2}}{ 1+3x+4x^{2}(1-x)+2\sqrt{2x(1+3x-2x^{3})}}, \end{aligned}$$

and consequently

$$h(x)= \frac{(2x-1)^{2}(2x+1)^{2}}{ 1+3x+4x^{2}(1-x)+2\sqrt{2x(1+3x-2x^{3})}}, $$

which is also positive for $x\in(0,1)$ and vanishes only if $x=1/2$. Thus $f(x)\ge0$ for $x\in(0,1)$ with equality if and only if $x=1/2$. The theorem is proved. □

Taking $\mu=3/4$ we obtain the next corollary.

### Corollary 3.9

*Let* $\triangle ABC$ *be a triangle*, *and let the lengths of sides opposite to angles* *A*, *B* *and* *C* *be denoted by* *a*, *b* *and* *c* *respectively*. *If* *r*, *R* *and* *F* *represent the inradius*, *the circumradius and the area of* $\triangle ABC$, *and if* $Q=(a-b)^{2}+(b-c)^{2}+(c-a)^{2}$ *then the following inequality holds*:

3.20 $$ 2F\sqrt{1+\frac{R}{r}}\le Q+2F\sqrt{\frac{6r}{R}} $$

*with equality if and only if the triangle is equilateral*. *Consequently*

$$a^{2}+b^{2}+c^{2}\le Q+4F\sqrt{1+ \frac{R}{r}} \le3Q+4F\sqrt{\frac{6r}{R}}. $$

In fact Theorem 3.8 allows us to give the following ‘parametric Finsler-Hadwiger inequality’.

### Corollary 3.10

*Let* *a*, *b*, *c*, *R*, *r* *and* *F* *be the side lengths of a triangle* $\triangle ABC$, *its circumradius*, *inradius and its area*, *respectively*, *and let* $Q=(a-b)^{2}+(b-c)^{2}+(c-a)^{2}$. *The following inequality*:

3.21 $$ a^{2}+b^{2}+c^{2}\le(1+2\lambda)Q+4F \biggl((1-\lambda)\sqrt{1+\frac {R}{r}}+\lambda\sqrt{\frac{6r}{R}} \biggr) $$

*holds for* $\lambda\ge0$, *and its reverse holds for* $\lambda\le-\frac{8\sqrt{6}+17}{95}\approx-0.38522$, *with equality if and only if the triangle is equilateral*.

### Proof

Note that (3.21) is equivalent to

$$a^{2}+b^{2}+c^{2}\le Q+4F\sqrt{1+ \frac{R}{r}} +2\lambda \biggl(Q+2F\sqrt{\frac{6r}{R}}-2F\sqrt{1+ \frac {R}{r}} \biggr). $$

So, according to Theorem 3.6 and Corollary 3.9, the proposed inequality (3.21) does hold for $\lambda\ge0$.

Now, using (3.18) and the fact that $4s^{2}=3(a^{2}+b^{2}+c^{2})-Q$ we see that Theorem 3.7 can be rephrased as follows:

$$4F \biggl( 4(1-\mu)\sqrt{1+\frac{R}{r}}-\sqrt{\frac{6r}{R}} \biggr)+(1-4\mu )Q\le(3-4\mu) \bigl(a^{2}+b^{2}+c^{2} \bigr) $$

for $\mu\ge5-2\sqrt{6}$. Now if we suppose that $\mu\in[5-2\sqrt{6},3/4)$ we conclude that

$$4F \biggl( \frac{4(1-\mu)}{3-4\mu}\sqrt{1+\frac{R}{r}}- \frac{1}{3-4\mu} \sqrt{\frac{6r}{R}} \biggr)+ \frac{1-4\mu}{3-4\mu}Q\le a^{2}+b^{2}+c^{2}. $$

Setting $\lambda=\frac{1}{4\mu-3}$ we get

$$4F \biggl( (1-\lambda)\sqrt{1+\frac{R}{r}}+ \lambda\sqrt{ \frac{6r}{R}} \biggr)+ (1+2\lambda)Q\le a^{2}+b^{2}+c^{2} $$

for $\lambda\in (-\infty,-\frac{1}{8\sqrt{6}-17} ]$, and the corollary is proved. □

### Remark 3.11

This inequality is the best of its kind in the sense that if $\lambda \in (-\frac{8\sqrt{6}+17}{95},0 )$ then there are triangles $\triangle ABC$ that satisfy (3.21) and others that violate it. Indeed, testing (3.21) with an isosceles triangle with side lengths $a=1$, $b=c=t\ge1/2$ for large *t* shows that the condition $\lambda\ge0$ is necessary for its validity. Testing its reverse for *t* near $1/2$ (but larger than $1/2$) shows that the condition $\lambda\le-\frac{8\sqrt{6}+17}{95}$ is necessary for the validity of the reverse.

### Remark 3.12

For $\lambda\le-\frac{8\sqrt{6}+17}{95}$ we have two different lower bounds for $a^{2}+b^{2}+c^{2}$ given by Theorem 3.6 and Corollary 3.10, respectively. Testing the difference with our famous isosceles triangle with side lengths $a=1$, $b=c=t\ge1/2$ shows that these two lower bounds are not comparable.

## Conclusion

In this work, we considered the problem of refining the Finsler-Hadwiger inequality and its reverse in the triangle. Several refinements are proposed and compared, and an optimal parametric refinement of this inequality and its reverse is proved.
